# The Italian Roma, Sinti and Travellers Health Project for equity in access to care

**DOI:** 10.1093/eurpub/ckac130.167

**Published:** 2022-10-25

**Authors:** A Diodati, MC Proietti, E Eugeni, R del Gaudio, LG Sisti, F Curtale

**Affiliations:** INMP, Rome, Italy

## Abstract

**Issue:**

In 2015, Italy approved the National Action Plan for Health for and with Roma, Sinti and Travellers (RSC); however, it is still not fully applied at a local level. The INMP (funded by the National Operational Programme for Social Inclusion), implemented the “Health Project - Promotion of strategies and tools for equity in access to health care for RSC”, aimed to support the implementation of the Plan by Local Health Authorities (ASL).

**Description:**

The project lasted from March 2019 to December 2021. INMP worked with 7 ASL to design interventions and develop tools to address RSC health needs. The SODA (Strategic Options Development and Analysis) method as a participatory process aimed at identifying strategies and models for the execution of the Plan among relevant stakeholders was used. Moreover, RSC communities’ engagement strategies and a community-based Proximity Public Health (PPS) approach were adopted in designing and developing health promotion interventions.

**Results:**

INMP performed 38 interviews with ASL health operators highlighting barriers and strategies in the local implementation of the Plan. Tools orienting to social and health services and health education materials for hard-to-reach groups were produced. A training course for 14 RSC facilitators and 5 training courses attended by over 200 NHS operators have been organized. ASL were supported in developing health promotion initiatives, based on the engagement of both local RSC communities and third sector entities.

**Lessons:**

The project has enabled the ASL to develop local protocols for the implementation of the Action Plan. In line with this capacity-building activity, the project trained both health operators and RSC facilitators fostering the dissemination of the PPS culture in designing and providing care for hard-to-reach groups. Given the positive results obtained, INMP is still collaborating with both ASL and the Third Sector on additional activities related to the Project in 2022.

**Key messages:**

- The project has strongly promoted the Proximity Public Health (PPS) approach and the enrollment of RSC communities in designing equity-oriented health interventions.
- The SODA methodology and the training of operators have resulted effective in capacitating ASL in the implementation of the Plan.

